# Meckel’s Diverticulum in a Five-Day-Old Neonate: An Unusual Case

**DOI:** 10.7759/cureus.114776

**Published:** 2026-08-19

**Authors:** Teresa T Costa

**Affiliations:** 1 Family Medicine, Unidade de Sáude da Ilha de São Miguel, Ponta Delgada, PRT

**Keywords:** meckel’s diverticulum, neonatal, neonatal care, neonatal metabolic acidosis, neonatal surgical intervention

## Abstract

Meckel’s diverticulum is a congenital anomaly of the gastrointestinal tract. We report the case of a five-day-old male infant who presented to the pediatric emergency department with lethargy, feeding refusal, grunting, bilious vomiting, and abundant bowel movements. Upon observation, he was hypotonic, jaundiced, and poorly perfused, with cool extremities and a soft abdomen. He was admitted to the neonatal intensive care unit, underwent nasogastric tube insertion, and was placed on nothing by mouth. The imaging studies were not conclusive. Subsequently, he progressed to metabolic acidosis. Due to suspicion of an acute surgical abdomen, he was transferred to a hospital with pediatric surgery capabilities. On day seven of life, he was diagnosed with Meckel’s diverticulum complicated by enteritis on exploratory laparotomy and underwent a segmental enterectomy with primary anastomosis. The patient was also diagnosed with late-onset sepsis caused by extended-spectrum beta-lactamase (ESBL)-negative *Escherichia coli*,and subsequent evaluation revealed a patent foramen ovale with a left-to-right shunt. During hospitalization, the patient remained hemodynamically stable, afebrile, and without respiratory distress, maintaining normal bowel and bladder functions. On the second day of hospitalization in the inpatient ward, the patient achieved full feeding autonomy. He was discharged on postoperative day 17. This case highlights that bilious vomiting in a neonate must always be considered a surgical emergency until proven otherwise. Prompt transfer to a pediatric surgery center can be lifesaving, particularly in geographically isolated regions. Although Meckel’s diverticulum is a rare entity in the neonate, it should remain in the differential diagnosis when initial imaging is non-diagnostic. Furthermore, despite the challenge of concurrent late-onset sepsis, targeted antibiotic therapy and appropriate clinical management led to the complete resolution of the infection caused by an ESBL-negative *E. coli* strain, ultimately resulting in an excellent postoperative outcome.

## Introduction

Meckel’s diverticulum, a remnant of the omphalomesenteric duct that normally involutes between the fifth and sixth weeks of human gestation, is the most common congenital anomaly of the gastrointestinal tract. It is a true diverticulum, containing all layers of the small bowel wall, and arises from the antimesenteric surface of the middle-to-distal ileum. While incomplete involution of the omphalomesenteric duct may result in a variety of anatomic patterns, the most common form is a persistent, unattached pouch [1].

In the general population, the prevalence of Meckel’s diverticulum has been estimated to be approximately 0.3% to 2.9%, although a systematic review found a prevalence of 1.2% among 31,499 autopsies in seven studies [2].

The “rule of twos” is the classic description and a useful mnemonic of the essential features of Meckel’s diverticulum. It states that Meckel’s diverticulum occurs in approximately 2% of the population with a male-to-female ratio of 2:1, is located within 2 feet of the ileocecal valve, and can be 2 inches in length, although, in practice, considerable anatomical and clinical variability has been described [3].

Meckel’s diverticulum is often clinically silent. Only 4% to 6% present with gastrointestinal bleeding or acute abdominal symptoms related to bowel obstruction, Meckel’s diverticulitis, or perforation. Between 25% and 50% of symptomatic patients present before 10 years of age. It is the most common cause of small intestinal bleeding in children, representing 31% of symptomatic Meckel’s diverticulum cases in this age group [4].

In the neonatal period, we found 20 case reports published in the last five years: seven presented with a perforated Meckel’s diverticulum, four with symptoms of intestinal obstruction, and two with hematochezia. Additionally, nine presented with other congenital anomalies. Overall, most cases had a favorable outcome.

Clinical features associated with an increased risk of developing symptoms from a Meckel’s diverticulum include age <50 years, male sex, diverticulum length greater than 2 cm, and presence of histologically abnormal tissue [5].

The differential diagnosis of Meckel’s diverticulum includes any etiology that can cause gastrointestinal bleeding, small bowel obstruction, or acute abdominal pain. There are no specific clinical features that reliably distinguish symptomatic Meckel’s diverticulum from other causes.

A definitive diagnosis of Meckel’s diverticulum is generally made through imaging studies or surgical exploration. A suspicion for an intermittent bleeding Meckel’s diverticulum can be investigated with a Meckel’s scan in hemodynamically stable patients, which identifies the presence of ectopic gastric mucosa within the diverticulum. In a 2023 systematic review and meta-analysis of 16 studies with 1,115 children, the combined sensitivity and specificity were 0.80 (95% confidence interval (CI) = 0.73-0.86) and 0.95 (95% CI = 0.86-0.98), respectively. A conventional or CT mesenteric arteriogram can be used to diagnose Meckel’s diverticulum with more brisk bleeding. Other abdominal symptoms are best investigated with a contrast-enhanced abdominopelvic CT scan [1].

Children with Meckel’s diverticulum are managed according to their clinical presentation. Symptomatic patients require surgical resection of the Meckel’s diverticulum. In asymptomatic children with incidental Meckel’s diverticulum identified on imaging studies, there are no data supporting a Meckel’s scan or proceeding to surgical resection [6]. For children eight years or younger with a normal-appearing Meckel’s diverticulum identified on abdominal exploration, although controversial, resection of the normal-appearing Meckel’s diverticulum is advised, as children have an increased lifelong risk for complications compared to adults [7].

## Case presentation

We report the case of a five-day-old male infant who was taken to the pediatric emergency department on an island without pediatric surgery services. The patient presented with lethargy, feeding refusal, grunting, bilious vomiting, and abundant bowel movements without blood. Symptoms began eight hours prior, following discharge from the hospital earlier that day, and the patient presented to the emergency department by the end of the evening.

The patient was a full-term neonate (38 weeks and 4 days) with appropriate-for-gestational-age birth anthropometrics (birth weight: 3,180 g (45th percentile); length: 49 cm (38th percentile); head circumference: 35 cm (62nd percentile)). The infant was delivered via cesarean section due to fetal bradycardia three hours following rupture of membranes, with a tight nuchal cord observed at delivery. Apgar scores were 7, 9, and 10 at 1, 5, and 10 minutes, respectively. Resuscitation required blow-by oxygen and mask positive pressure ventilation, to which the patient responded well. The newborn spent the first five days of life rooming-in with the mother, receiving two cycles of phototherapy for ABO isoimmunization and infant formula due to unsuccessful breastfeeding.

On observation, he was hypotonic, jaundiced, poorly perfused, with cool extremities and a soft abdomen. He was admitted to the neonatal intensive care unit, underwent nasogastric tube insertion, and was placed on nothing by mouth. The gastric aspirate contained ~20 mL of partially digested milk and bile.

Initially, on day six, his laboratory workup revealed a hemoglobin level of 16.8 g/dL, a white blood cell count of 1,630/µL (neutrophils: 600/µL), and a platelet count of 179,000/µL. Renal function and liver function tests were within normal limits. C-reactive protein was elevated at 1.97 mg/dL (Table 1).

**Table 1:** Laboratory investigation on admission and follow-up.

Parameter	Day 6 (admission)	Day 6 (9-hour follow-up)	Day 7	Reference range
Hemoglobin	16.8 g/dL	16.7 g/dL	12.9 g/dL	13.4–19.9 g/dL
White blood cells	1,630/µL	3,980/µL	10.950/µL	9,000–30,000/µL
Neutrophils	600/µL	1,700/µL	7,540/µL	1,000–20,000/µL
Platelets	179,000/µL	179,000/µL	174,000/µL	150,000–450,000/µL
Creatinine	0.63 mg/dL	0.62 mg/dL	0.39 mg/dL	0.30–1.00 mg/dL
Urea	—	31 mg/dL	33mg/dL	10–30 mg/dL
C-reactive protein	1.97 mg/dL	6.02 mg/dL	134.1 mg/dL	<1.0 mg/dL
Procalcitonin	—	383.2 ng/mL	—	<0.5 ng/mL
Prothrombin time	—	26 seconds	—	10.6–16.2 seconds
International normalized ratio	—	2.05	—	0.9–1.3
Activated partial thromboplastin time	—	67.4 s	—	27.0–79.0 seconds
D-dimer	—	3,496 ng/mL	—	<500 ng/mL
Fibrinogen	—	330 mg/dL	—	150–370 mg/dL
Aspartate aminotransferase	28 U/L	41 U/L	25 U/L	<34 U/L
Alanine aminotransferase	14 U/L	—	23 U/L	10–49 U/L
Gamma-glutamyl transferase	114 U/L	86 U/L	58 U/L	17.0–175 U/L
Alkaline phosphatase	194 U/L	149 U/L	96 U/L	83–248 U/L
Total bilirubin	10.9 mg/dL	9.06 mg/dL	7.31 mg/dL	<14.6 mg/dL
Conjugated bilirubin	0.95 mg/dL	0.83 mg/dL	0.43 mg/dL	0.3–0.5 mg/dL
Albumin	—	2.2 g/dL	2.6 mg/dL	2.8–4.4 g/dL
Calcium	—	8.4 mg/dL	8.9 mg/dL	8.5–10.6 mg/dL
Phosphorus	—	7.9 mg/dL	4.9 mg/dL	4.2–9.0 mg/dL

**Figure 1:**
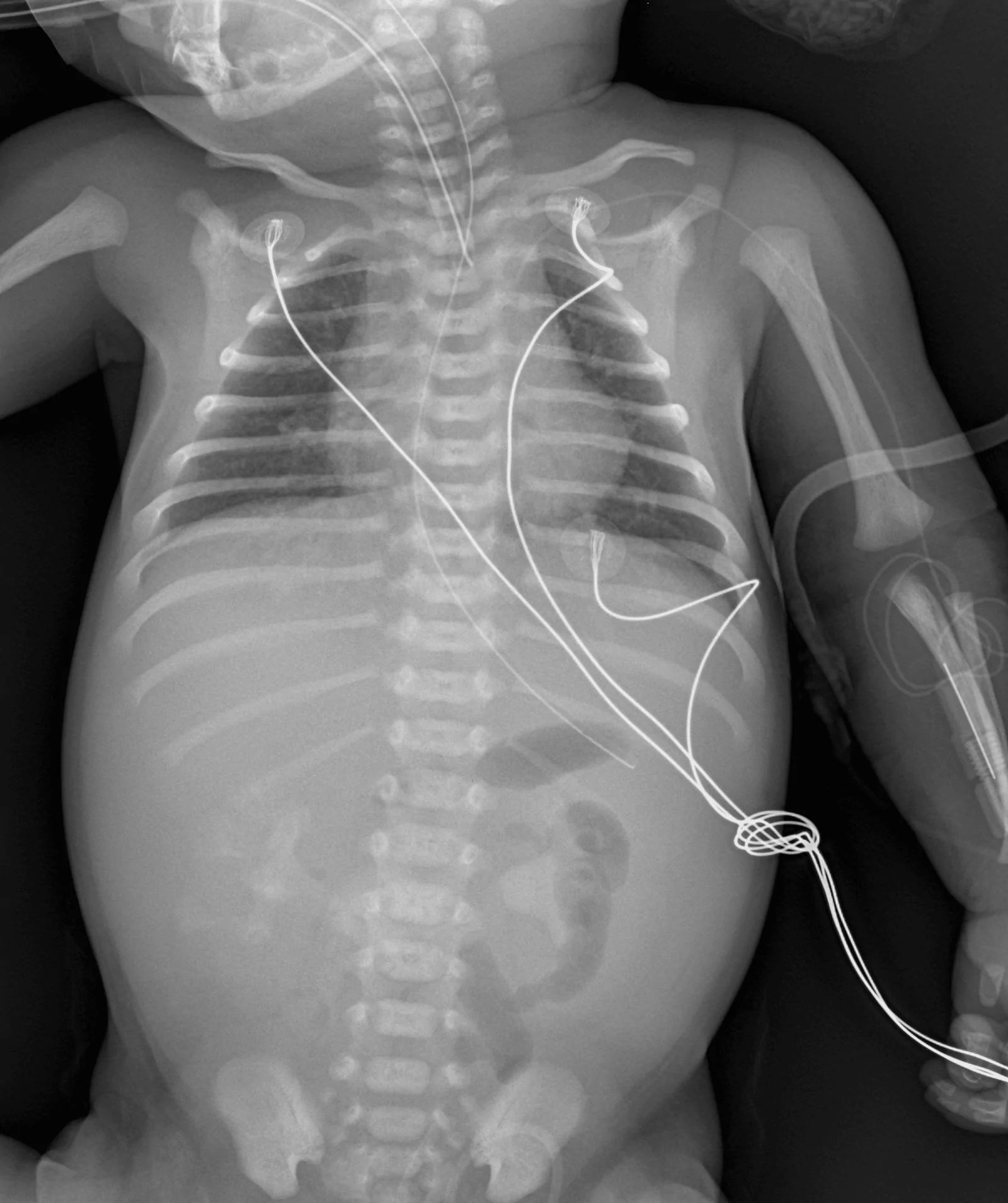
Abdominal anterior-posterior radiograph showing little visible air in the bowel loops.

**Figure 2:**
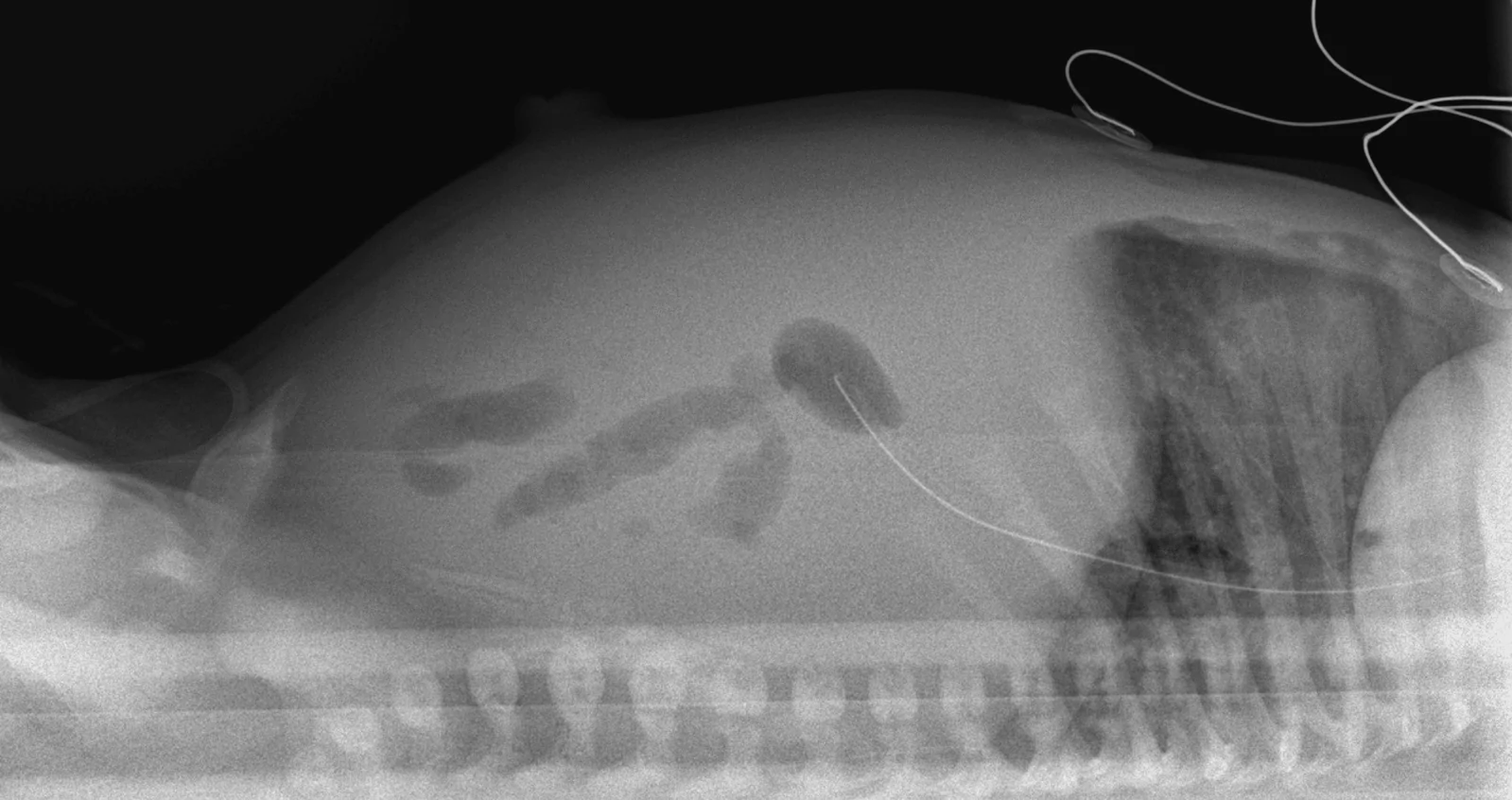
Lateral abdominal radiograph showing little visible air in bowel loops.

An abdominal radiograph showed little visible air in the bowel loops (Figures 1, 2). An abdominal ultrasound showed inter-loop fluid, thin and sparse perigastric septations, and well-positioned mesenteric vessels, not confirming malrotation with volvulus.

Abdominal and pelvic contrast-enhanced CT with intravenous iodinated contrast demonstrated non-distended intestinal loops with a normal distribution, no significant wall thickening, preserved mucosal enhancement, and no signs of pneumatosis intestinalis. There were no signs of volvulus or the “Whirlpool sign” of mesenteric vessels, and no images suggestive of intussusception. A few mesenterico-juxtaparietal gas bubbles were identified, predominantly in the posterior periduodenal region, making an extraluminal location possible; their distribution remained unchanged following intragastric air insufflation via the nasogastric tube. Moderate-volume ascites was observed across all abdominal recesses, suggesting peritonitis (possibly subacute in evolution). Slight cardiomegaly was also observed. The initial differential diagnosis included late neonatal sepsis, malrotation with volvulus, and necrotizing enterocolitis. Due to a mean arterial pressure of ~40 mmHg and low urine output, a dopamine infusion was initiated at ~8 µg/kg/minute.

Follow-up laboratory evaluation at nine hours revealed an increase in inflammatory markers: white blood cell count of 3,980/µL (neutrophils: 1,700/µL), platelets of 179,000/µL, prothrombin time of 26 seconds, international normalized ratio of 2.05, activated partial thromboplastin time of 67.4 seconds, D-dimer of 3,496 ng/mL, C-reactive protein of 6.02 mg/dL, procalcitonin of 363 ng/mL, low albumin of 2.2 g/dL, and calcium of 8.4 mg/dL (Table 1). Due to suspected infection, empiric antibiotic therapy was initiated with cefotaxime, gentamicin, and metronidazole.

Subsequently, the patient developed metabolic acidosis. Due to suspected acute surgical abdomen, supported by clinical deterioration, gasless abdomen, and moderate-volume ascites suggesting bowel ischemia and peritonitis, he was evacuated on day six by the Air Force to a mainland hospital with pediatric surgery capabilities. However, transfer was prolonged due to logistical delays, and the patient arrived at the destination 11 hours after the decision to transfer.

On arrival on day seven, the patient was re-evaluated with a repeat abdominal ultrasound, which did not confirm intestinal malrotation with volvulus. Instead, it revealed diffuse parietal thickening of intestinal loops, most prominent in the small intestine of the upper quadrants (3.5 mm), but also affecting the colon (4.5 mm). The ileocecal appendix appeared to have contiguous parietal thickening, but without significant luminal ectasia. There was no significant dilatation of the various bowel segments, which overall showed hypomotility. A small amount of free fluid was present in the upper and lower recesses of the abdominal cavity. The patient also underwent a transfontanellar ultrasound, which was normal, and a lumbar puncture, which was sterile.

Follow-up laboratory evaluation on day seven revealed a marked increase in inflammatory markers: white blood cell count of 10,950/µL (neutrophils: 7,540/µL), platelets of 174,000/µL, and C-reactive protein of 134.1 mg/dL (Table 1).

On day seven, due to a lack of clinical improvement, ascites, and markedly increased inflammatory markers, the patient underwent exploratory laparotomy. An enlarged periumbilical incision was performed. A citrine-colored peritoneal effusion was noted, along with abundant fibrin deposits. Exteriorization of the entire bowel was performed, revealing a diverticulum located approximately 10 cm from the ileocecal valve on the antimesenteric border of the ileum, consistent with a Meckel’s diverticulum, with signs of local infection and dense adhesions to the mesentery. A segmental enterectomy of approximately 1 cm, incorporating the diverticulum, was performed. End-to-end ileo-ileal anastomosis was constructed in two layers, with a continuous inner layer and an interrupted outer layer. A small mesenteric defect was closed, and abundant peritoneal lavage was performed. The abdominal wall was closed in layers.

He was also diagnosed with late-onset sepsis caused by extended-spectrum beta-lactamase (ESBL)-negative *Escherichia coli*, identified in blood cultures obtained on day six of life. Subsequently, a patent foramen ovale with a left-to-right shunt was diagnosed during an evaluation for cardiomegaly, which had been incidentally found on the abdominal CT scan.

Gross examination of the surgical specimen revealed an intestinal diverticular formation consistent with Meckel’s diverticulum, measuring 3.9 cm in length and 1.1 cm in maximum thickness. Microscopic evaluation confirmed a diverticular formation with endoluminal hemorrhage, lined by small intestinal mucosa. Heterotopic gastric mucosa or pancreatic parenchyma was not observed. The wall exhibited marked edema and interstitial hemorrhage, with a fibrinogranulocytic exudate on the serosal surface, suggestive of a prior perforation. The non-diverticular intestinal wall segment was unremarkable.

During hospitalization, the patient remained hemodynamically stable, afebrile, and without respiratory distress, maintaining normal bowel and bladder functions. On the second day of hospitalization in the inpatient ward, the patient achieved full feeding autonomy. Laboratory values normalized during hospitalization, and follow-up blood cultures yielded no growth. The patient was discharged on postoperative day 17 (day of life 24), with a prescription for cholecalciferol at a dose of 666 IU/day.

**Figure 3:**
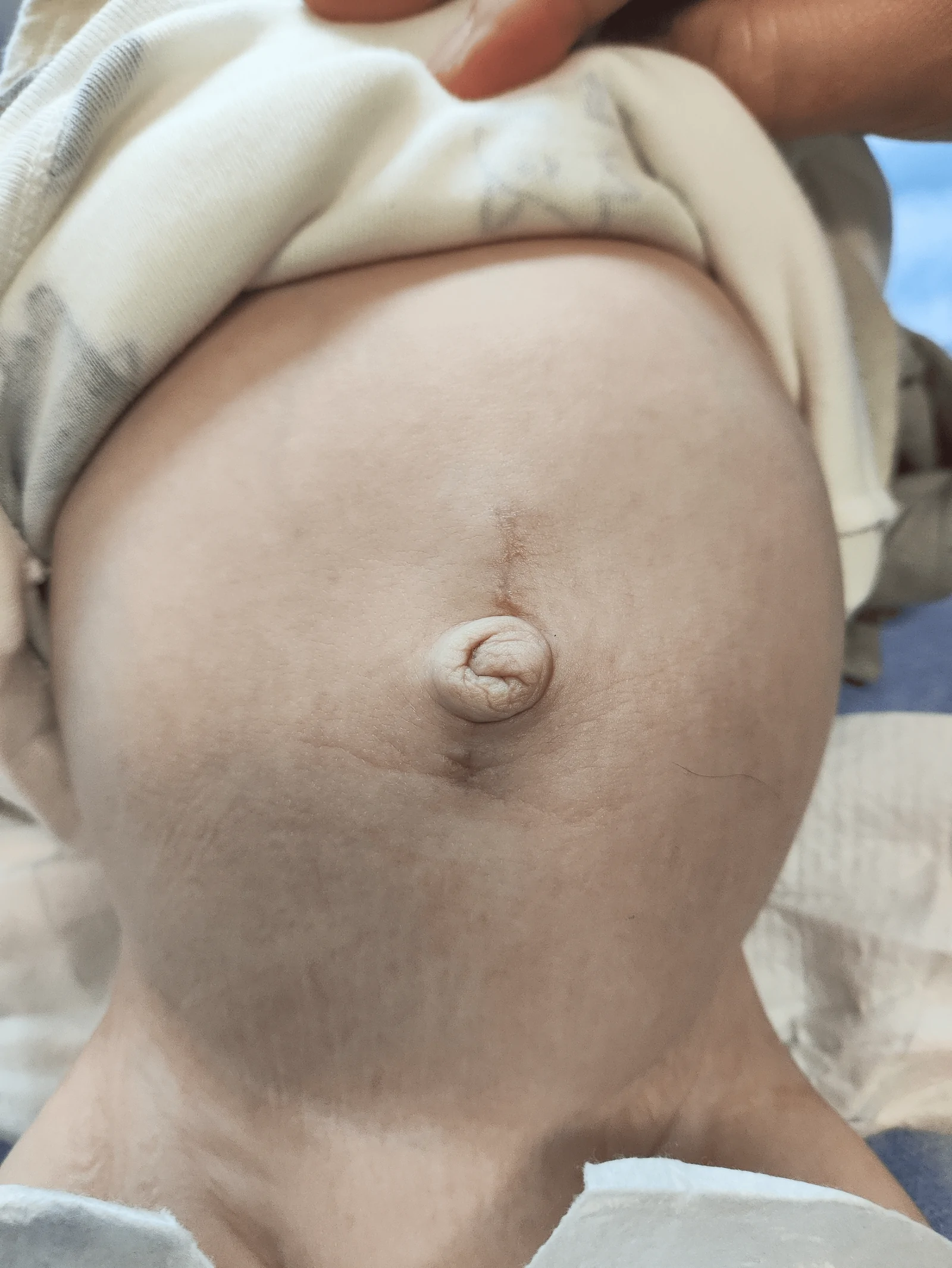
Appearance of the infant’s abdominal surgical scar during follow-up at 37 days of life.

The patient was subsequently evaluated by his family physician at 37 days of life. On examination, his weight was 3,290 g, indicating poor weight gain (an increase of 110 g from birth weight), which was expected due to surgery and sepsis. Physical findings were otherwise unremarkable, except for a grade I/VI systolic murmur on cardiac auscultation and a healing abdominal surgical scar (Figure 3).

At a follow-up visit at 55 days of life, his weight had increased to 3,915 g, reflecting an average weight gain of 34.7 g/day. The patient was awaiting a pediatric cardiology appointment.

## Discussion

The clinical presentation of this five-day-old male patient constituted a catastrophic neonatal emergency. In a remote or island setting lacking immediate access to pediatric surgical subspecialties, the medical team was faced with a critical dilemma: distinguishing a rapidly progressive surgical abdomen from severe medical mimics. This patient had both.

In neonatologists’ clinical practice, bilious emesis in a newborn is considered an intestinal obstruction, specifically intestinal malrotation with midgut volvulus, until proven otherwise [8]. The clinical presentation in this patient (feeding refusal and vomiting and initial evacuation of abundant bowel movements) was suggestive of this entity.

In contrast, symptomatic Meckel’s diverticulitis in the immediate neonatal period is an exceedingly rare entity that poses a major diagnostic challenge. While Meckel’s diverticulum classically presents in older children with painless lower gastrointestinal bleeding or intussusception, presentation within the first week of life frequently mimics midgut volvulus or necrotizing enterocolitis due to acute perforation, peritonitis, or intestinal obstruction [9,10].

Because early-stage surgical abdomens in neonates present with profound systemic symptoms, they are frequently misdiagnosed as neonatal sepsis or septic shock. Severe bacterial infections (e.g., *E. coli* or Group B *Streptococcus*) routinely cause paralytic ileus, abdominal distension, and emesis, which may become bile-stained in advanced stages [9,10]. In this patient, the coexistence of late-onset *E. coli* sepsis and Meckel’s diverticulitis was believed to be secondary to bacterial translocation driven by intestinal inflammation or perforation, given that the pathological report suggested prior perforation of the Meckel’s diverticulum.

Liaqat et al. [11] reviewed 59 case reports of perforated Meckel’s diverticulum in neonates published between 1938 and 2021. Most patients (84.7%) presented within the first week of life, 39.0% were born prematurely, 79.7% were male, and 52.5% had associated congenital anomalies. Preoperative imaging rarely indicated the underlying cause of pneumoperitoneum, with only one case suspected preoperatively and the remainder diagnosed intraoperatively during laparotomy. In 73.0% of cases, surgeons performed segmental resection with primary intestinal anastomosis. Histopathological assessment revealed no ectopic gastric mucosa in 44 (74.6%) cases, suggesting another possible etiology of the perforation. Three patients died before intervention and were diagnosed at autopsy. Overall, seven (11.9%) deaths occurred; the outcome was not reported in one (1.7%) case, and 51 (84.7%) patients were successfully discharged.

Other critical medical differentials include inborn errors of metabolism and the salt-wasting form of congenital adrenal hyperplasia. Accumulation of toxic metabolites or acute mineralocorticoid deficiency can trigger profound metabolic acidosis, dehydration, lethargy, and intractable vomiting, perfectly mimicking an acute surgical crisis [12].

Initial management must run in parallel with diagnostic attempts based on immediate cessation of enteral feeds, insertion of a nasogastric tube for continuous bowel decompression, aggressive fluid resuscitation to address distributive or hypovolemic shock, and prompt administration of broad-spectrum empiric antibiotics to cover both primary neonatal sepsis and potential bacterial translocation across an ischemic intestinal barrier.

Due to surgery and sepsis, initial weight loss was expected during the period of fasting and metabolic recovery. Following the introduction and tolerance of full enteral nutrition, a standard weight gain of 15-30 g/day was anticipated, with an excellent long-term prognosis owing to the near-total preservation of bowel length.

The management of this case highlights the profound impact of geographical barriers on neonatal outcomes. In an isolated setting without pediatric surgical coverage, the priority shifts from definitive diagnosis to aggressive stabilization and rapid, effective medical evacuation. In this patient, transfer was prolonged due to logistical delays, underscoring the need to review protocol procedures and optimize the process.

Ultimately, this case underscores that while sepsis mimics must be managed aggressively, mechanical obstructions, such as volvulus or complicated Meckel’s diverticulitis, require urgent surgical intervention. When managing an infant with bilious emesis in a resource-limited setting, maintaining a high index of suspicion and expediting transfer are paramount to preventing irreversible bowel necrosis and death.

## Conclusions

This case highlights that bilious vomiting in a neonate must always be considered a surgical emergency until proven otherwise, even in the presence of suspected or confirmed sepsis. Prompt transfer to a pediatric surgery center can be lifesaving, particularly in geographically isolated regions. Although Meckel’s diverticulum is a rare entity during the neonatal period, it should remain in the differential diagnosis when initial imaging is non-diagnostic. Furthermore, despite the challenge of concurrent late-onset sepsis, targeted antibiotic therapy and appropriate clinical management led to the complete resolution of the infection caused by an ESBL-negative *E. coli* strain, ultimately resulting in an excellent postoperative outcome and a return to normal neonatal development.
